# Comment on ‘Quantification of Skeletal Muscle at the First Lumbar Level for Prognosis in Amyotrophic Lateral Sclerosis’ by Cao et al.

**DOI:** 10.1002/jcsm.70038

**Published:** 2025-07-26

**Authors:** Josef Finsterer

**Affiliations:** ^1^ Neurology Department Neurology & Neurophysiology Centre Vienna Austria

**Keywords:** amyotrophic lateral sclerosis, axial muscles at L1, disease outcome, disease severity, muscle area and density

We were interested to read the article by Cao et al. on a cohort study on the validity of L1‐level skeletal and paraspinal muscle density and area on chest CT (SMD, SMA, PMA and PMD) and subcutaneous area and density (SFA and SFD) to assess severity and outcomes in 102 patients with sporadic and familial amyotrophic lateral sclerosis (SALS and FALS) [1]. SMD, SMA, PMA and PMD were lower in ALS patients, and SFA and SFD were higher than in control subjects [1]. The ALS Functional Rating Scale‐Revised (ALSFRS‐R) increased with increasing SMA, skeletal muscle index (SMI) and PMA [1]. SMD, SMA and PMD significantly influenced ALS survival [1]. The study is remarkable, but some ambiguities should be clarified.

The first point is that ALS is currently diagnosed according to the Gold Coast criteria [2]. What is the reason that the revised Awaji‐Shima criteria were used to diagnose definite, probable, and possible ALS? How many of the included patients did not have ALS when the Gold Coast criteria were applied?

The second point is that ALS can occur in the limbs as well as the trunk, suggesting that skeletal and paraspinal muscle mass may be lower in patients with bulbar onset than in those with limb ALS. We should know how many of the included patients had limb‐onset ALS and how many had bulbar ALS and whether there was a difference in outcome parameters between these two groups.

The third point is that the chest CT was performed only once and was not repeated after a certain time. To assess whether muscle parameters actually worsened with disease progression and how this affected survival and outcome, it would have been useful to repeat the imaging studies.

The fourth point is that the outcome parameters can also depend heavily on the condition of the lumbar spine [3]. Therefore, we should know how many patients had scoliosis, lumbar malalignment, chondrosis, osteochondrosis, spondylosis, spondylarthrosis and osteoarthritis of the uncovertebral joints, spinal canal stenosis or myelopathy, and how this affects the outcome parameters.

The fifth point is that muscle mass may also depend on diet and nutritional status. Since ALS is often associated with dysphagia, it is conceivable that hypoproteinemia contributes to muscle loss and is more pronounced in patients with dysphagia than in patients without dysphagia. Was there a difference in outcome parameters between these two groups? In how many patients was a percutaneous gastrostomy (PEG) implanted?

The sixth point is that muscle mass may also depend on respiratory function, especially whether a patient is hypoxic or not. Therefore, we should know how many patients require oxygen supplementation, non‐invasive ventilation, continuous positive airway pressure (CPAP) or a tracheostomy. A disadvantage in this respect is that only 40 patients had a pulmonary function test [1].

The seventh point is that no tests of within‐ or between‐observer variability were performed to assess the stability of the CT measurements.

The eighth point is that six of the included patients had FALS [1]. Therefore, it would be interesting to know whether the outcome parameters differed between SALS and FALS patients.

Until the aforementioned limitations are addressed, skeletal and paraspinal muscle area and density at the L1 level can only be used with reservation as a measure of severity and outcome in ALS patients.

## Conflicts of Interest

The author declares no conflicts of interest.

## Data Availability

All data are available from the corresponding author.
